# ﻿A new species of *Sterculia* (Malvaceae) from Vietnam

**DOI:** 10.3897/phytokeys.227.101754

**Published:** 2023-05-29

**Authors:** Cam Nhung Kieu, Duc Binh Tran, Ngoc Han Le, Thi Hoan Duong, Thu Ha Bui, Thu Thuy Nguyen, Hong Quang Bui, The Bach Tran

**Affiliations:** 1 Graduate University of Science and Technology, Vietnam Academy of Science and Technology, 18 Hoang Quoc Viet, Cau Giay, Ha Noi, Vietnam Graduate University of Science and Technology, Vietnam Academy of Science and Technology Ha Noi Vietnam; 2 Department of Botany, Institute of Ecology and Biological Resources, Vietnam Academy of Science and Technology, 18 Hoang Quoc Viet, Cau Giay, Ha Noi, Vietnam Institute of Ecology and Biological Resources, Vietnam Academy of Science and Technology Ha Noi Vietnam; 3 Faculty of Biology, Hanoi National University of Education, 136, Xuan Thuy, Cau Giay, Ha Noi, Vietnam Hanoi National University of Education Ha Noi Vietnam

**Keywords:** Malvaceae, Sterculia, Tay Nguyen, Vietnam

## Abstract

A new species of *Sterculia* from Vietnam – *S.konchurangensis* – is described, illustrated, and compared with the similar *S.lanceolata*. *S.konchurangensis* differs from *S.lanceolata* by the length of the petiole (7.0–9.5 vs. 25–35 mm), shape of the leaf blade (obovate or elliptic vs. elliptic, lanceolate or elliptic-lanceolate), length of the leaf blade (6–8 *vs.* 9–20 cm), and length of the calyx lobe (11–12.5 vs. 4–6 mm). A diagnostic key of the 22 *Sterculia* species occurring in Vietnam is also provided.

## ﻿Introduction

The genus *Sterculia* L. (Malvaceae Juss.) comprises 100–200 species mainly distributed in tropics and subtropics of both hemispheres, most abundant in Asian tropics (see e.g., Ya et al. 2007; POWO 2023; WFO 2023). The genus is characterized by having leaves simple, flowers unisexual, androgynophore present, staminodes at top of androgynophore in whorl around base of carpels (Ya et al. 2007). In Vietnam, 22 species of *Sterculia* have been recorded so far (Gagnepain 1910; Nguyen et al. 1980; Pham 1999; Chamlong 2001; Nguyen 2003; Newman et al. 2007; Ya et al. 2007; POWO 2023).

During a botanical survey of the Gia Lai province (the Central Highlands of Vietnam) in 2022, plants referred to the genus *Sterculia* were observed in a primary evergreen forest in the Kon Chu Rang Nature Reserve. After comparing the collected specimens with others preserved in various herbaria, and by consulting relevant literature, we reached the conclusion that Vietnamese population represent a new species for science which is here described and illustrated. We also provide a key to the species of *Sterculia* that are now known to occur in Vietnam.

## ﻿Materials and methods

The morphology of the new species were observed on both living plants and herbarium specimens. Branches, leaves and (functionally female) flowers (lf, f. fl.) of type materials are stored at the Institute of Ecology and Biological Resources (HN) and the Institute of Tropical Biology (**VNM**) (acronyms follow Thiers 2023[continuously updated]). The conservation status of the new species was assessed according to the guidelines of the International Union for Conservation of Nature (IUCN 2019).

## ﻿Taxonomy

### ﻿ Sterculia konchurangensis

Taxon classificationPlantaeMalvalesMalvaceae

C.N.Kieu, D.B.Tran & B.H.Quang sp. nov.

21F51AEE-A235-512F-B95D-7EFF61C743A4

urn:lsid:ipni.org:names:77320206-1

Figs 1 , 2 , 3

#### Type.

Vietnam. Tay Nguyen: Gia Lai province, Kon Chu Rang reserve, 1016 m a.s.l., 19 June 2022 (lf, f. fl.), *Bui Hong Quang et al. BHQ 576* (holotype HN!, isotypes: HN!, VNM!).

**Figure 1.**
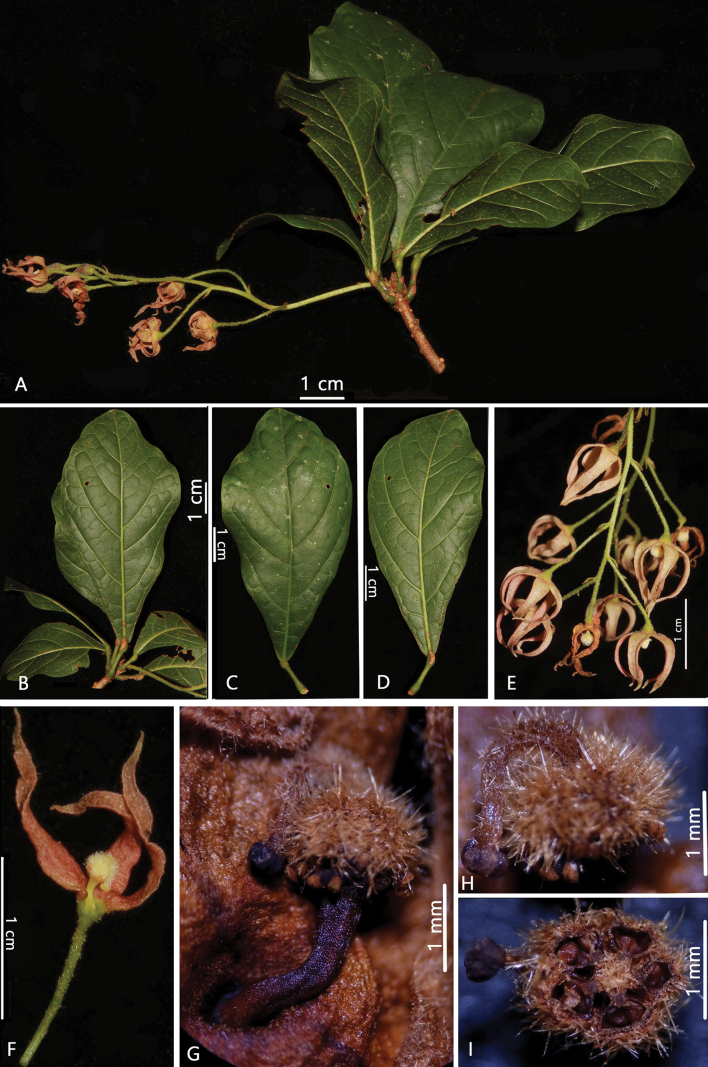
*Sterculiakonchurangensis* C.N.Kieu, D.B.Tran & B.H.Quang **A** flowering branch **B, C, D** leaf blade **E** inflorescence **F** open flower showing androgynophore, ovary, style, stigma **G** androgynophore, staminodes, ovary, style, stigma **H** ovary, style, stigma **I** section ovary, style, stigma (Photos by D.B Tran).

#### Diagnosis.

*Sterculiakonchurangensis* is most similar to *S.lanceolata* Cav. due to the number of veins on each side of midrib, length of inflorescence, deeply divided calyx, globose, hairy ovary and curved style. They are separated by morphology of leaf blades (obovate or elliptic in *S.konchurangensis vs*. elliptic, lanceolate or elliptic-lanceolate in *S.lanceolata*); petioles are shorter (7.0–9.5 mm vs. 25–35 mm in *S.lanceolata*), leaf blades are shorter (6–8 cm vs. 9–20 cm in *S.lanceolata*) and calyx lobes are longer (11–12.5 mm vs. 4–6 mm in *S.lanceolata*).

**Figure 2.**
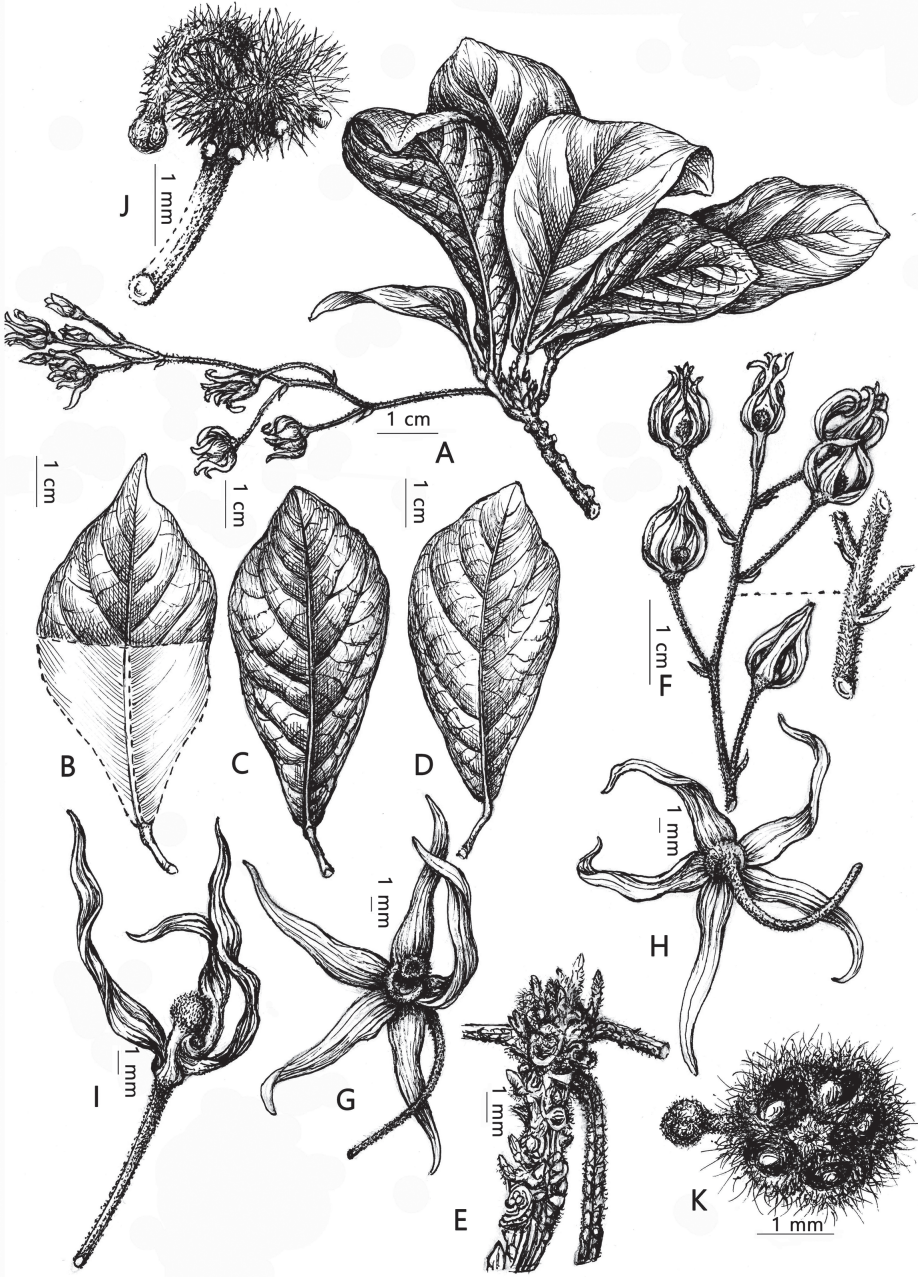
*Sterculiakonchurangensis* C.N.Kieu, D.B.Tran & B.H.Quang **A** flowering branch **B, C, D** leaf blade **E** apex branches and stipules **F** inflorescence **G** flower **H** flower **I** open flower showing androgynophore, ovary, style, stigma **J** androgynophore, ovary, style, stigma, staminodes **K** section ovary, style, stigma (Drawn by Le Kim Chi).

#### Description.

***Shrubs***, ca. 3 m tall. ***Branches*** gray brown. **Leaves** alternate, apically clustered; ***petiole*** 7.0–9.5 mm long, glabrous, base and apex of petiole swollen; leaf blade simple, entire, glabrous, obovate, elliptic, base attenuate, apex shortly acuminate or obtuse, 6–8 × 3–4 cm, both surfaces glabrous; ***lateral veins*** 6–8 on each side of midrib. ***Stipules*** linear, ca. 1–2 mm long. ***Inflorescence*** slender, racemose, axillary, 8 cm long, few-12 flowered. ***Pedicel*** slender, 9.5–12.0 mm long, densely villous. ***Flowers*** 5-merous, functionally unisexual. Flowers: ***Flower bud*** lanceolate, 6.3 × 2.3 mm. ***Calyx*** divided almost to base, 5-lobed, pink, adaxial surface nearly glabrous to sparsely pubescent, abaxial surface pubescent; ***tube*** 1.3–1.7 mm long; ***lobes*** linear-lanceolate, 11–12.5 × 2.5–2.8 mm. ***Petals*** absent. ***Androgynophore*** slender, curved, ca. 2 mm long, glabrous. ***Staminodes*** at top of androgynophore in whorl around base of carpels; ***anthers*** of staminodes ovate, 0.22 × 0.18 mm. ***Carpels*** 5; ***ovary*** globose, densely pubescent, 1.3–1.8 mm in diameter; ***style*** curved, 2–3 mm long, sparsely pubescent; ***stigma*** glabrous, 0.3–0.6 mm in diameter. ***Fruits and seeds*** not observed.

**Figure 3.**
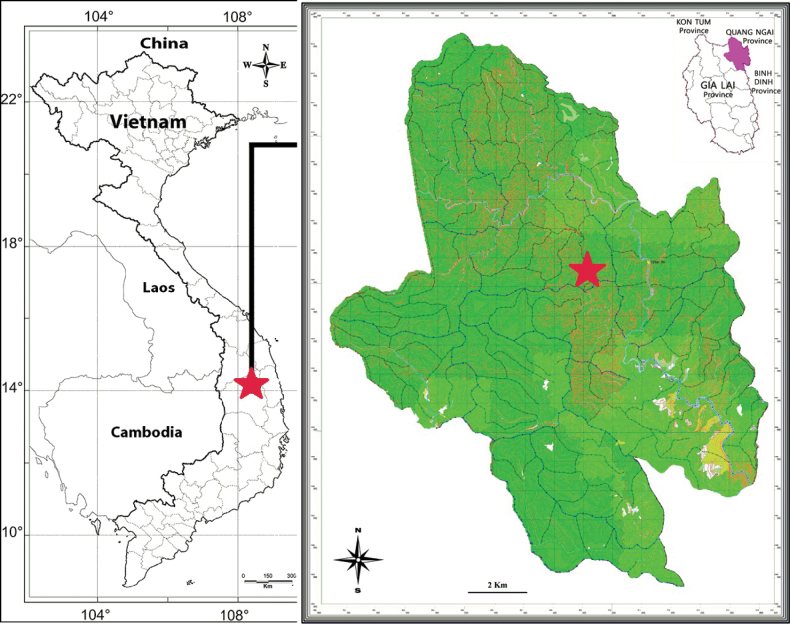
Map indicating the type locality of *Sterculiakonchurangensis* C.N.Kieu, D.B.Tran & B.H.Quang (Made by D.B Tran & T.B. Tran).

#### Etymology.

The specific epithet refers to the type locality, Kon Chu Rang reserve in Vietnam.

#### Distribution and ecology.

*Sterculiakonchurangensis* is only found in Vietnam, Gia Lai province, Tay Nguyen area, Kon Chu Rang reserve where it grows in primary evergreen forest at an altitude of 1016 m a.s.l. Flowering time is June; fruiting time is unknown.

#### Conservation status.

Data Deficient (DD; IUCN 2019). *Sterculiakonchurangensis* is known only from the type locality within Kon Chu Rang reserve. A comprehensive botanical survey of the *Sterculia* has not been carried out to date.

#### Discussion.

*Sterculiakonchurangensis* is morphologically similar to *S.lanceolata* Cav. due to the number of veins on each side of midrib, length of the inflorescence, deeply divided calyx, globose, hairy ovary, and curved style; furthermore, to be noted that both species have the same flowering time (June). However, *S.konchurangensis* differs from *S.lanceolata* by the morphology of length of petiole, shape of leaf blade, size of leaf blade and length of calyx lobe.

The identified key of 23 species of *Sterculia* in Vietnam was constructed. *Sterculiakonchurangensis* differs from 22 species of *Sterculia* by having some characters such as simple, entire, glabrous leaves and length of petiole (less than 12 mm long). In addition, the comparison with the similar species (*S.lanceolata*) confirms that *S.konchurangensis* is a new species. Diagnostic characters separating the two species are listed in Table 1.

**Table 1.** Morphological differences between *S.lanceolata* and *S.konchurangensis*.

Characters	S.lanceolata	S.konchurangensis
Length of petiole (mm)	25–35	7.0–9.5
Shape of leaf blade	elliptic, lanceolate or elliptic-lanceolate	obovate or elliptic
Size of leaf blade (cm)	9–20 × 3.5–8.0	6–8 × 3–4
Length of calyx lobe (mm)	4–6	11–12.5

### ﻿Key to the species of *Sterculia* in Vietnam

**Table:** 

1	Leaves palmately compound	**2**
–	Leaves simple	**3**
2	Calyx purple-red, ca. 12 mm long, divided nearly to base	***S.foetida***
–	Calyx white, ca. 6 mm long, divided to 1/2 of the total length	***S.pexa***
3	Leaf blade lobed	**4**
–	Leaf blade not lobed	**5**
4	Seeds 2 per follicle	***S.hypochroa***
–	Seeds 6–7 per follicle	***S.thorelii***
5	Basal veins 3–7	**6**
–	Basal veins absent	**9**
6	Leave base shallow cordate	***S.stigmarota***
–	Leave base not shallow cordate	**7**
7	Lateral veins 5–6 on each side of midrib	**8**
–	Lateral veins 7–10 on each side of midrib	***S.principis***
8	Seeds 1–2 per follicle	***S.lissophylla***
–	Seeds 3–6 per follicle	***S.chrysodasys***
9	Leave pubescent	**10**
–	Leaves glabrous	**14**
10	Leaves obovate or oblanceolate	**11**
–	Leaves elliptic-oblong	**12**
11	Lateral veins 16–24 on each side of midrib	***S.hymenocalyx***
–	Lateral veins less than 12 on each side of midrib	***S.parviflora***
12	Petiole 5–7 cm long	***S.radicans***
–	Petiole less than 4 cm long	**13**
13	Leaves silver hairy beneath	***S.pierrei***
–	Leaves rufous hairy beneath	***S.tonkinensis***
14	Petiole < 12 mm long	***S.konchurangensis***
–	Petiole > 15 mm long	**15**
15	The upper parts of the lateral veins connected	**16**
–	The upper parts of the lateral veins not connected	**20**
16	Lateral veins more than 6 pairs	**17**
–	Lateral veins 5–6-paired	***S.cochinchinensis***
17	Lateral veins less than 12 pairs	**18**
–	Lateral veins 12–15 paired	***S.henryi***
18	Petiole 2.5–8.0 cm long	**19**
–	Petiole 1–2 cm long	***S.hyposticta***
19	Calyx reddish, divided almost to base	***S.lanceolata***
–	Calyx dark brown, calyx united at the bottom 1/3	***S.aberrans***
20	Calyx 3.5–4.5 mm long	***S.gracilipes***
–	Calyx longer than 5 mm	**21**
21	Androgynophore longer than calyx tube	***S.bracteata***
–	Androgynophore shorter than calyx tube	**22**
22	Petiole 2–5 cm long	***S.monosperma***
–	Petiole 7–10 cm long	***S.scandens***

## Supplementary Material

XML Treatment for Sterculia konchurangensis

## ﻿Additional information

### Author ORCIDs

Cam Nhung Kieu https://orcid.org/0009-0002-7480-1030

Duc Binh Tran https://orcid.org/0000-0001-6658-0739

Ngoc Han Le https://orcid.org/0000-0003-2657-5091

Thi Hoan Duong https://orcid.org/0000-0002-4298-5720

Thu Ha Bui https://orcid.org/0000-0001-6971-0105

Thu Thuy Nguyen https://orcid.org/0000-0002-0921-5652

Hong Quang Bui https://orcid.org/0000-0001-6878-7514

The Bach Tran https://orcid.org/0000-0003-3798-5969

### Data availability

All of the data that support the findings of this study are available in the main text or Supplementary Information.
